# The Women’s wellness after cancer program: a multisite, single-blinded, randomised controlled trial protocol

**DOI:** 10.1186/s12885-017-3088-9

**Published:** 2017-02-03

**Authors:** Debra Anderson, Charrlotte Seib, Dian Tjondronegoro, Jane Turner, Leanne Monterosso, Amanda McGuire, Janine Porter-Steele, Wei Song, Patsy Yates, Neil King, Leonie Young, Kate White, Kathryn Lee, Sonj Hall, Mei Krishnasamy, Kathy Wells, Sarah Balaam, Alexandra L. McCarthy

**Affiliations:** 1 Menzies Health Institute Queensland, Griffith University, Menzies Health Institute Queensland, Parklands Drive, Southport, Queensland 4215 Australia; 2 Institute of Health and Biomedical Innovation, Queensland University of Technology, Brisbane, Queensland Australia; 3 University of Queensland, Brisbane, Queensland Australia; 4 Royal Brisbane and Women’s Hospital, Brisbane, Queensland Australia; 5 University of Notre Dame, Perth, Western Australia Australia; 6 St John of God Murdoch Hospital, Perth, Western Australia Australia; 7 Choices Cancer Support Program, Wesley Hospital, Brisbane, Queensland Australia; 8 Shanghai Ocean University, Shanghai, China; 9 University of Sydney, Sydney, New South Wales Australia; 10 Royal Prince Alfred Hospital, Sydney, New South Wales Australia; 11 University of California San Francisco, California, USA; 12 Central Queensland University, Brisbane, Queensland Australia; 13 Peter MacCallum Cancer Institute, Melbourne, Victoria Australia; 14 Breast Cancer Network Australia, Melbourne, Victoria Australia; 15 Division of Cancer Services, Princess Alexandra Hospital, Brisbane, Queensland Australia

**Keywords:** Cancer, Women, health-related quality of life, Menopausal symptoms, Modifiable lifestyle factors

## Abstract

**Background:**

Despite advances in cancer diagnosis and treatment have significantly improved survival rates, patients post-treatment-related health needs are often not adequately addressed by current health services. The aim of the Women’s Wellness after Cancer Program (WWACP), which is a digitised multimodal lifestyle intervention, is to enhance health-related quality of life in women previously treated for blood, breast and gynaecological cancers.

**Methods:**

A single-blinded, multi-centre randomized controlled trial recruited a total of 330 women within 24 months of completion of chemotherapy (primary or adjuvant) and/or radiotherapy. Women were randomly assigned to either usual care or intervention using computer-generated permuted-block randomisation. The intervention comprises an evidence-based interactive *iBook* and journal, web interface, and virtual health consultations by an experienced cancer nurse trained in the delivery of the WWACP. The 12 week intervention focuses on evidence-based health education and health promotion after a cancer diagnosis. Components are drawn from the American Cancer Research Institute and the World Cancer Research Fund Guidelines (2010), incorporating promotion of physical activity, good diet, smoking cessation, reduction of alcohol intake, plus strategies for sleep and stress management. The program is based on Bandura’s social cognitive theoretical framework. The primary outcome is health-related quality of life, as measured by the Functional Assessment of Cancer Therapy-General (FACT-G). Secondary outcomes are menopausal symptoms as assessed by Greene Climacteric Scale; physical activity elicited with the Physical Activity Questionnaire Short Form (IPAQ-SF); sleep measured by the Pittsburgh Sleep Quality Index; habitual dietary intake monitored with the Food Frequency Questionnaire (FFQ); alcohol intake and tobacco use measured by the Australian Health Survey and anthropometric measures including height, weight and waist-to-hip ratio. All participants were assessed with these measures at baseline (at the start of the intervention), 12 weeks (at completion of the intervention), and 24 months (to determine the level of sustained behaviour change). Further, a simultaneous cost-effectiveness evaluation will consider if the WWACP provides value for money and will be reported separately.

**Discussion:**

Women treated for blood, breast and gynaecological cancers demonstrate increasingly good survival rates. However, they experience residual health problems that are potentially modifiable through behavioural lifestyle interventions such as the WWACP.

**Trial registration:**

The protocol for this study was registered with the Australian and New Zealand Clinical Trials Registry, Trial ID: ACTRN12614000800628, July 28, 2014.

## Background

While the overall incidence of female cancers is increasing, advances in diagnosis and treatment have significantly improved survival rates. In some cases, this has transformed cancer from an often fatal condition to a chronic and often curable disease (for example, early breast malignancies) [1]. Although survival rates are improving, survival can involve a number of treatment-related health problems including ovarian failure [2], weight gain [3], cognitive alterations [4], and fatigue [5]. For women after cancer, these physical and psychological sequelae can be severe and frequently adversely affect their quality of life [2].

Recent studies indicate that many survivorship-related health problems can be modified through lifestyle practices such as good diet, greater physical activity, and participation in screening programs, though cancer survivors do not necessarily adopt these behaviours [6, 7]. According to two large American studies, few people treated for cancer met recommendations for smoking cessation, alcohol minimisation, physical activity, or fruit and vegetable intake [6, 8]. Conversely, our pilot work indicates that many cancer survivors are highly motivated to protect their health and are more likely to take up beneficial lifestyle and risk reduction strategies if they are targeted towards the end of or soon after completion of treatment [9, 10].

Australian health services and cancer support organisations do not often provide structured health promotion programs to support cancer survivors to minimise lifestyle-related health risks once they have ceased active treatment, despite the public and personal health benefits that these would provide. While a range of survivorship programs in recent years have recognised the chronic nature of cancer, most are narrowly targeted on specific symptoms and not based on sound chronic disease self-management principles and the management of risk through lifestyle [11]. The need for support is particularly acute among rural and outer metropolitan survivors, who have restricted access to face-to-face services due to cost, time, geographical, and other barriers. Novel approaches to promote beneficial lifestyle practices in women after cancer, such as the WWACP, have the potential to improve quality of life and reduce chronic disease risk.

The aim of this randomised controlled trial (RCT) is to determine the efficacy and cost effectiveness of a multimodal, digitised lifestyle intervention — The Women’s Wellness after Cancer Program or WWACP. The WWACP aims to enhance health-related quality of life in women previously treated for breast, gynaecological, or blood cancers. The hypotheses are that, compared to usual care, women undertaking the WWACP intervention will:

1. Demonstrate higher scores of HRQoL irrespective of their place of residence.
2. Be more likely to have a body mass index (BMI) within recommended healthy weight range (e.g. BMI 20–25, waist circumference < 80 cm)
3. Show greater long term adherence to diet, exercise, sleep, alcohol, and smoking recommendations.
4. Evaluate if the WWACP provides better value for money than the current usual care approaches (reported elsewhere).

## Methods

**Fig. 1:**
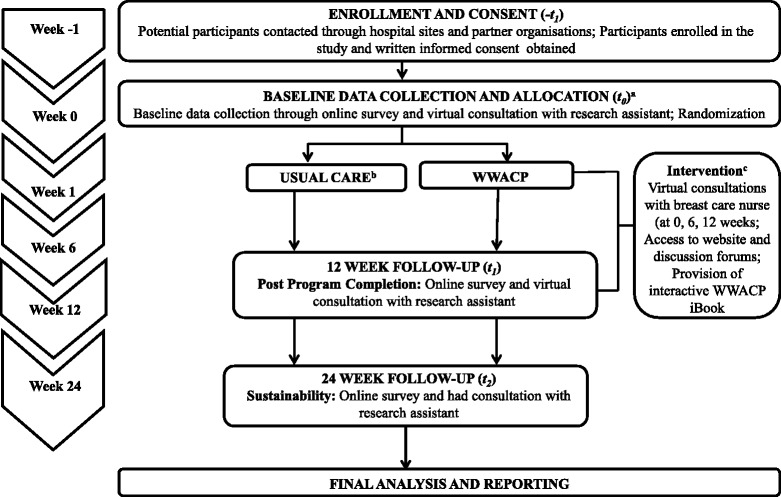
Flowchart for the WWACP study. Notes: ^**a**^ All participants completed a structured online questionnaire and virtual consultation with a research assistant at baseline (*t0*), 12 weeks (*t1*) and 24 weeks (*t2*); ^**b**^ The control group received general information only; ^**c**^ The intervention group received intervention materials and three virtual consultations with a breast cancer nurse at baseline (*t0*), 6 weeks (mid-intervention), and 12 weeks (*t1*)

This multi-centre, single-blinded, randomized controlled 12-week trial involved five hospitals (public and private) and consumer groups serving women from metropolitan and non-metropolitan areas throughout Australia. The study was funded by a National Health and Medical Research Council (NHMRC) Partnership Grant (APP1056856). It received ethical approval from the relevant Institutional Review Boards of participating sites before commencing the trial. Details of the trial are further illustrated in Fig. 1.

### Sampling and recruitment

The study population included women aged 18 and treated for breast, blood or gynaecological cancer within the previous 24 months. These cancers were chosen as they comprise approximately 46% of all female cancers in Australia in 2007 [1] and their treatment is associated with numerous ongoing health complaints. These problems include menopausal symptoms, osteopenia and osteoporosis, obesity, diabetes, cognitive alterations, peripheral neuropathy and cardiac health consequences. Good evidence indicates that many of these comorbidities can be prevented or mitigated by consistent engagement in health promoting activities and weight reduction [10, 12–15], which are the foci of this intervention.

Participants were recruited by clinicians from five hospital sites: Princess Alexandra Hospital, Royal Brisbane and Women’s Hospital, St John of God Murdoch Hospital, Peter MacCallum Cancer Institute, and the Choices Cancer Support Program; and through newsletters, emails and websites of three consumer groups (the National Breast Cancer Foundations’ Register4, CanSpeak Queensland, and the Breast Cancer Network of Australia).

Inclusion criteria included completion of chemotherapy (primary or adjuvant) and/or radiotherapy within the previous 24 months for breast, blood, or gynaecological cancer; English proficiency; and access to an Apple computer and or iPad (due to interactive book’s comparability). Participants were excluded if they had metastatic or advanced cancer, inoperable or active loco-regional disease, were on maintenance chemotherapy for blood cancers.

### Randomisation

The RCT comprises two treatments (intervention vs usual care) and three time points for data collection; baseline (t0), 12 weeks (t1) and 24 weeks (t2). The procedure for random allocation occurred after baseline data collection and was performed by a member of the research team independent of recruitment or data collection. Permuted-block randomisation was used, with the seed for the random number generator determined by entering the number of treatments, and the numbers of patients per specific block (i.e., with two treatment groups and block sizes ranging from 2–8). The exact sequence was generated using algorithms [16, 17] available at http://www.randomization.com.

### Intervention

This multi-modal intervention was designed for delivery via an e-health enabled platform. It comprises virtually-delivered health professional consultations, an *interactive web interface* (including podcasts), an interactive electronic book (*iBook*) which provides detailed intervention instructions and supports participants to log relevant health and lifestyle information into a journal.

The intervention targets health education and health promotion incorporating Australian and international recommendations for physical activity, diet, smoking cessation and minimising alcohol intake; as well as strategies to manage sleep and stress, menopausal symptoms, and sexual problems over a 12 week period (see Tables 1 and 2 for further detail). The timing and application of these strategies were tailored to meet patient’s individual goals and functional capacities.

**Table 1:** WWACP Intervention Content and Delivery Strategies

Week	Delivery Strategies	Content
1	Virtual consultation delivered by specialist cancer nurse	Physical activity, and healthy eating messages; goal setting; education; motivational interviewing; development of tailored health education and individualised plan and goals. Observational weight, and self-measured height, waist/hip circumference measures
2	Phone coaching, Journal, Book, health education material/website	Review plan and goals; develop a personal action plan; identify barriers; self-monitoring
3	Phone coaching, Journal, Book, and website	Relapse prevention; coaching, feedback, motivational interviewing and self-monitoring.
4,12	Journal/website/SMS/e-mail	Mobile phone text message every week based on program messages; news update every four weeks; motivational messages sent as women reach set goals.
6,12	Virtual RN consultation	Review of plan and goals; coaching; relapse prevention; motivational interviewing; biophysical measurements. Observational weight and self-measured height, waist/hip circumference measures
1,12, 24	Data collection by RA	Observational, weight, waist/hip circumference measures and on-line questionnaire

**Table 2:** The Women’s Wellness after Cancer Program targeted behaviours [40]

Targeted Behaviours	Rationale/Evidence
Body Fatness: Be as lean as possible within the normal weight range, avoid weight gain and increases in waist circumference	Maintenance of a healthy weight may be one of the most important ways to protect against cancer recurrence and other common chronic diseases, including hypertension, stroke, type 2 diabetes and coronary heart disease [40]
Physical Activity: Be moderately physically active, equivalent to brisk walking, for at least 30 min per day; As fitness improves, aim for 60 min or more of moderate, or for 30 min or more of vigorous, physical activity every day	Physical activity of longer duration or greater intensity is more beneficial; All forms of physical activity protect against some cancers, as well as against weight gain, overweight, and obesity [40]
Diet: Eat mostly foods of plant origin; limit consumption of energy-dense foods; avoid sugary drinks, limit intake of red meat and avoid processed meat	An integrative approach to the evidence shows that most diets that are protective against cancer are mainly made up from foods of plant origin; Consumption of energy-dense foods and sugary drinks contributes to obesity; An integrated approach to the evidence also shows that many foods of animal origin are nourishing and healthy if consumed in modest amounts.
Alcohol: If alcoholic drinks are consumed limit consumption to no more than one drink per day	The evidence on cancer justifies a recommendation not to drink alcoholic drinks. Other evidence shows that modest amounts of alcoholic drinks are likely to reduce the risk of coronary heart disease [40]

The *iBook* is structured around four chronological steps: changing lifestyle; establishing healthy habits; maintaining health for illness prevention; and becoming independent. More specifically, Step 1 provides essential information about a number of topics that form the basis of the 12-week program. Participants receive practical advice about how to incorporate this information into their lifestyle. Step 2 encourages participants to apply their new lifestyle habits to the areas of healthy weight, strong bones and hormonal change. Step 3 presents information about maintaining health for illness prevention. Step 3 emphasises a healthy heart, diabetes prevention, and cancer screening so that participants can manage their treatment-related risks for chronic disease in a proactive way. Finally, Step 4 focusses on developing independence and sustaining healthy lifestyle changes after the 12-week program.

The *interactive website* provided healthy living support and home monitoring of measurable health indicators, which are downloaded to the study team. The website incorporates access to the downloadable *iBook*; educational podcasts and factsheets; a weekly exercise planner and schedule; and a community message board.

*Virtual consultations* were provided by experienced cancer nurses trained in the delivery of the WWACP, via skype or FaceTime at weeks 0, 6, and 12 (see Table 3 for further detail). These consultations included discussion and health education about physical activity, healthy eating, stress, sleep menopause and sexuality after cancer and strategies to promote intervention adherence. The women had v physical and psychological capabilities, the nurses therefore worked with them to set realistic goals within the woman’s capacity.

**Table 3:** Consultation nurse delivery strategies and content

Week	Delivery strategies	Content
1	Virtual consultation delivered by specialist cancer nurse: Phone coaching, iBook, health education material, website and email	Introduction to website and IBook. Physical activity, and healthy eating messages; goal setting; education; motivational interviewing; development of tailored health education and individualized plan and goals. Discuss healthy weight measures and associated risk factors i.e. BMI, waist/hip ratio. Discuss menopause, stress, sleep and other concerns. Discuss appropriate screening.
3 (email)		Email to check progress
6		Review plan and goals; Discuss personal action plan; identify barriers, self-monitoring
12		Reviews of plan and goals; coaching; relapse prevention; motivational interviewing; biophysical measurements; review observational weight and self-measured height, waist/hip circumference measures.

### Standard care

Participants allocated to the standard care group received general information from their usual health professionals during clinic visits about the management of all symptoms, including general advice about exercise, diet, tobacco and alcohol abstinence, plus information about support services. No specific or individual advice was provided, as per usual practice.

### Quality assurance

This study was conducted in accordance with the International Conference of Harmonisation Guideline E6 – Good Clinical Practice (ICH-GCP) recommendations for clinical trials. Regular auditing of the adequacy and effectiveness of internal controls was undertaken0of risk management and compliance frameworks by the independent Clinical Trial Manager of the lead university.

To ensure the reliability of the anthropometrical measures and survey data collected by research assistants (RAs), standard operating procedures were developed. RAs also received a one-day training workshop prior to commencing data collection and were audited periodically by the research manager.

The fidelity of the nurse consultations was enhanced by ensuring that the trial nurses, who were experienced in cancer care, were also trained in the use of the intervention. Nurses received a self-directed protocol manual and DVD, and participated in a full day skills development session delivered by members of the research team. To ensure adherence to study protocols, peer review of virtual consultations was conducted by the clinical manager and nurses also completed a checklist at the end of each session to indicate the strategies used.

### Measurements

Primary and secondary endpoints were measured at three time points (baseline, post-intervention or 12, and 24 weeks after the end of the trial) through a self-completed electronic survey or through RA data collection. Table 4 outlines the timing and mode of administration for each instrument. Data collectors were blinded to the group allocation of the participants.

**Table 4:** Outcome measures, instruments, modes of administration, and time points of the study

Measures	Instruments		Time point		Mode of administration	
		*t*_*0*_ (baseline)	*t*_*1*_ (12 weeks)	*t*_*2*_ (24 weeks)	Online survey	RA data collection
Background information						
Socio-demographics						
Medical and surgical history		X	Assesses changes in medical/surgical history, and medications since baseline			X
Cancer diagnosis and treatment		X				X
Medications		X				X
Subjective health indicators						
HRQoL	FACT G [18]	X	X	X	X	
	SF – 36 [41]	X	X	X	X	
Depression	CES – D [42]					
Anxiety	Zung SAS [43]					
Sexuality						
Sexual function	FSFI [44]	X	X	X	X	
Exercise self-efficacy	ESE [45]	X	X	X	X	
Dietary self-efficacy	DSE [45]	X	X	X	X	
Menopausal symptoms						
Menopausal symptoms	GCS [23]	X	X	X	X	
Modifiable lifestyle factors						
Diet	FFQ [46, 47]	X	X	X		X
Physical Activity	IPAQ [48]					
Sleep	PSQI [27]					
Waist and hip circumference		X	X	X		X
Cost effectiveness evaluation						
Calendar of costs incurred		X	X	X		X

*FACT G* Functional Assessment of Cancer Therapy – General, *SF* – 36, Short Form 36, *CES* – D, Center for Epidemiologic Studies Depression Scale, *Zung SAS* Zung self-rating anxiety scale, *FSFI* Female Sexual Function Index, *ESE* Exercise Self-Efficacy, *DSE* Dietary Self-Efficacy, *GCS* Greene Climacteric Scale; *FFQ* Food Frequency Questionnaire, *IPAQ* International Physical Activity Questionnaire, *PSQI* Pittsburgh sleep quality index

#### Primary endpoint

The primary outcome measure was health-related quality of life as determined by the Functional Assessment of Cancer Therapy-General (FACT-G) [18], a 28-item instrument used extensively to assess satisfaction with the treatment relationship, physical, functional, social, and emotional well-being, and overall quality of life (QoL). Psychometric testing has shown good internal consistency (Cronbach alphas: physical sub-scale, 0.82; functional sub-scale, 0.80; social sub-scale, 0.69; emotional sub-scale, 0.72; and total score, 0.89), test-retest reliability (0.88, 0.84, 0.82, 0.82, 0.92 respectively), and validity [18].

There is little consensus over the minimal clinically important difference (MCID) in health-related quality of life scores that constitutes a meaningful difference for participants (either beneficial or harmful), with MCID in the FACT-G total scores ranging from 4–8 points [19–21]. Using a distribution-based approach, a 6 point or greater improvement in the FACT-G total scores (between *t*_*0*_ and *t*_*1*_) was considered a minimal clinically important change for women in the study [19–22].

#### Secondary endpoints

Changes in several secondary outcome measures were also assessed with the following instruments:

1. Menopausal symptoms were measured using the standard Greene Climacteric Scale© [23], a 21-item scale that assesses self-reported vasomotor symptoms, somatic symptoms, psychological symptoms (anxiety and depression), and sexual function [23]. This scale has consistently demonstrated good psychometric properties and has been used in population-based and clinical samples in a variety of locales [23, 24].
2. Anthropometric measures were collected by RAs using standard protocols for the measurement of height and weight (from which BMI was derived), waist-to-hip ratio, and percentage of body fat. BMI was grouped according to the WHO International Classification of adult weight [25] (i.e., <18.5 being underweight, between 18.5 - 24.9 being in the normal weight range, and ≥ 30 being obese).
3. Alcohol and tobacco use was assessed using several questions from the Australian Health Survey, a population–based survey designed that assesses the past and current patterns of consumption among the Australian population aged 18 and over [26].
4. Habitual dietary intake (including greater detail of alcohol consumption) was monitored through the Food Frequency Questionnaire (FFQ) [27, 28]. The FFQ covers dietary intake of cereal foods, sweets and snacks, dairy products, meats and fish, fruit, vegetables, and alcoholic beverages [27, 28] and is one of few dietary intake measures that minimises day-to-day variability by assessing usual dietary intake in the past 12-months.
5. Physical activity was measured using the validated International Physical Activity Questionnaire Short Form (IPAQ-SF) [29]. The short version of this instrument is recommended for use in national and regional surveillance systems [30]. It assesses three types of activity (walking, moderate-intensity activities and vigorous-intensity activities), which are all targeted behaviours within the intervention.
6. Sleep activity and quality were measured using the Pittsburgh Sleep Quality Index (PSQI), a 19-item self-report instrument with well-established reliability and validity [27]. The PSQI includes seven sub-scales (subjective sleep quality, sleep latency, sleep duration, habitual sleep efficiency, sleep disturbances, use of sleeping medication, and daytime dysfunction) and an overall summary score and has demonstrated utility in both clinical and research settings [27].

### Data analysis

#### Sample size calculations

*A priori* power analysis [28, 31] determined the minimum sample size required for each group is 268, with a total sample size of 536 (calculation based on: standardised mean difference (SMD) = 0.65, 90% power, 95% confidence interval; 25% non-response). However, delays in ethical approval across sites and slower than anticipated recruitment meant the initial calculation were revised. Thus *A posteriori* power analysis were performed and revealed that 250 participants (125 per group) were required to detect a standardised mean difference (SMD) of 0.6 or greater [32, 33] in the primary outcome measure (FACT-G total). Calculations were based on achieving 80% power, with a type I error of 5% (two-tailed) to detect a 5.7 change in the standard deviation of the FACT-G total (M = 76.2, SD = 14.3 from pilot study [10]), and 25% non-adherence (10% lost to follow-up and 15% non-response).

#### Primary analysis

All statistical data will be analysed using Statistical Package for the Social Sciences (SPSS)® version 23 [34] and STATA 13 [35] statistical packages in adherence with the CONSORT reporting guidelines [36]. Baseline measures and participant characteristics will be initially compared to assess for imbalances and differences between groups while the primary endpoint (HRQoL) will examine within- and between-group differences in FACT-G scores over the study period. Logistic regression models will examine predictors of quality of life (including baseline quality of life), age and sociodemographic variables. This includes exploring the rurality of the women according to the classifications of highly accessible, accessible, moderately accessible, remote, and very remote location suggested by the Accessibility/Remoteness Index of Australia (ARIA) [37]. Linear mixed models (LMM) will analyse between-group changes and changes over time in secondary outcomes such as energy, macronutrient and micronutrient intake, body fatness, menopausal symptoms, levels of physical activity, alcohol, and smoking.

## Discussion

Recent research indicates that women diagnosed with blood, breast, and gynaecological cancers have a distinct set of health needs after treatment. For example, among breast cancer survivors menopausal symptoms, infertility, fatigue, lymphedema, and osteoporosis can persist after treatment and are likely to have a significant and negative affect on ongoing health and wellness [38]. In many instances, the health system does not adequately address these health issues and is struggling to support the increasing number of cancer survivors in the population who subsequently develop treatment-related chronic diseases [39].

This study seeks to trial the effectiveness of a digitised, multimodal lifestyle intervention for the management of treatment-induced late health effects in women after cancer. We used an e-health enabled platform to reduce accessibility issues associated with cost, time, geographical, and other constraints.

Close-out will occur November 2016, at which time data will be prepared for the longitudinal data analysis. The findings from this study will contribute to evidence-based information about the utility and benefits of structured health promotion activities for women after cancer.

## Abbreviations

ARIA: Accessibility/Remoteness index of Australia
BCNA: Breast cancer network Australia
BMI: Body mass index
FACT-G: Functional assessment of cancer therapy-general
FFQ: Food frequency questionnaire
HREC: Human research ethics committee
HRQoL: Health-related quality of life
HRT: Hormone replacement therapy
IPAQ: International physical activity questionnaire
KWC: Kim Walter’s choices
LMM: Linear mixed models
NHMRC: National health and medical research council
QALYs: Quality of adjusted life years
QoL: Quality of life
RACGP: Royal Australian college of general practitioners
RAs: Research assistant’s
RCT: Randomized controlled trial
SMD: Standardized mean difference
SPSS: Statistical package for the social sciences
WHO: World health organisation
WWACP: Women’s wellness after cancer program
WWP: Women’s wellness program
