# Erratum to: The Women’s wellness after cancer program: a multisite, single-blinded, randomised controlled trial protocol

**DOI:** 10.1186/s12885-017-3189-5

**Published:** 2017-03-16

**Authors:** Debra Anderson, Charrlotte Seib, Dian Tjondronegoro, Jane Turner, Leanne Monterosso, Amanda McGuire, Janine Porter-Steele, Wei Song, Patsy Yates, Neil King, Leonie Young, Kate White, Kathryn Lee, Sonj Hall, Mei Krishnasamy, Kathy Wells, Sarah Balaam, Alexandra L. McCarthy

**Affiliations:** 1 Menzies Health Institute Queensland, Griffith University, Menzies Health Institute Queensland, Parklands Drive, Southport, Queensland 4215 Australia; 2 Institute of Health and Biomedical Innovation, Queensland University of Technology, Brisbane, Queensland Australia; 3 University of Queensland, Brisbane, Queensland Australia; 4 Royal Brisbane and Women’s Hospital, Brisbane, Queensland Australia; 5 University of Notre Dame, Perth, Western Australia Australia; 6 St John of God Murdoch Hospital, Perth, Western Australia Australia; 7 Choices Cancer Support Program, Wesley Hospital, Brisbane, Queensland Australia; 8 Shanghai Ocean University, Shanghai, China; 9 University of Sydney, Sydney, New South Wales Australia; 10 Royal Prince Alfred Hospital, Sydney, New South Wales Australia; 11 University of California San Francisco, California, USA; 12 Central Queensland University, Brisbane, Queensland Australia; 13 Peter MacCallum Cancer Institute, Melbourne, Victoria Australia; 14 Breast Cancer Network Australia, Melbourne, Victoria Australia; 15 Division of Cancer Services, Princess Alexandra Hospital, Brisbane, Queensland Australia

## Erratum

After the publication of this work [1] errors were noticed in the Methods section of the Abstract. In the line “A single-blinded, multi-centre randomized controlled trial recruited a total of 330 women within 24 months of completion of chemotherapy (primary or adjuvant) and/or radiotherapy”, the number 330 should be corrected to 351.

In the line “All participants were assessed with these measures at baseline (at the start of the intervention), 12 weeks (at completion of the intervention), and 24 months (to determine the level of sustained behaviour change)”, 24 months should be corrected to 24 weeks.
